# PP01 Pulsed Field Ablation Versus Thermal Ablation For Paroxysmal Atrial Fibrillation: A Literature Review

**DOI:** 10.1017/S0266462325102213

**Published:** 2025-12-29

**Authors:** Anna Pereira, Henrique Tortele, Maiara Antonio, Murilo Contó

## Abstract

**Introduction:**

Atrial fibrillation (AF) is the most common sustained arrhythmia. Thermal catheter ablation (radiofrequency or cryoablation) is a standard treatment but risks complications like pulmonary vein stenosis. Pulsed field ablation (PFA), a non-thermal alternative, offers selective myocardial targeting through irreversible electroporation. This literature review evaluated PFA’s safety and efficacy compared to thermal ablation to support its incorporation into the Brazilian Supplementary Health sector.

**Methods:**

Electronic databases (MEDLINE via PubMed, the Cochrane Library, LILACS, and Embase) were systematically searched through to March 2024, identifying 421 titles after excluding duplicates. Following two screening stages, six articles were fully read. Data were integrated, and duplicates were removed using Rayyan artificial intelligence tool. Eligible studies included systematic literature reviews (SLRs) and randomized controlled trials (RCTs) comparing PFA with both thermal ablation methods (radiofrequency and cryoablation). Observational studies were included as additional evidence. Evidence quality was assessed using the GRADE tool by outcome. The risk of bias was evaluated with the revised Cochrane risk-of-bias tool for RCTs and AMSTAR-2 for SLRs.

**Results:**

One SLR with meta-analysis (moderate quality) showed that PFA had shorter procedure times without significant differences in rates of periprocedural complications or recurrent atrial fibrillation. In another study by the same authors (low quality), significantly fewer periprocedural complications were found in the PFA group versus thermal ablation (p=0.001). Another SLR with meta-analysis (high quality) reported higher durability percentages for pulmonary vein isolation with PFA. The ADVENT trial (no critical bias concerns, moderate certainty of evidence) showed PFA’s non-inferior efficacy and safety relative to thermal ablation, with less pulmonary vein narrowing. Observational studies highlighted PFA’s favorable learning curve.

**Conclusions:**

PFA had a favorable safety profile and non-inferior efficacy compared with thermal ablation. It resulted in fewer periprocedural complications and greater durability in pulmonary vein isolation. Its favorable learning curve suggests PFA can be efficiently integrated into clinical practice with minimal procedural challenges. These findings support PFA as a promising alternative for treating paroxysmal AF in Brazil.

